# Association between physical activity and mortality in end-stage kidney disease: a systematic review of observational studies

**DOI:** 10.1186/s12882-021-02407-w

**Published:** 2021-06-18

**Authors:** Pedro Martins, Elisa A. Marques, Diogo V. Leal, Aníbal Ferreira, Kenneth R Wilund, João L. Viana

**Affiliations:** 1 Research Center in Sports Sciences, Health Sciences and Human Development, CIDESD, University Institute of Maia, ISMAI, Maia, Portugal; 2 Fresenius Medical Care, NephroCare, Lisbon, Portugal; 3 Curry Cabral Hospital, University Hospital Centre of Central Lisbon, Lisbon, Portugal; 4 Nova Medical School, Lisbon, Portugal; 5 Department of Kinesiology and Community Health, University of Illinois at Urbana-Champaign, Champaign, Illinois USA

**Keywords:** Chronic Kidney Disease, Hemodialysis, Survival, Hospitalization, Physical Activity

## Abstract

**Background:**

End-stage Kidney Disease patients have a high mortality and hospitalization risk. The association of these outcomes with physical activity is described in the general population and in other chronic diseases. However, few studies examining this association have been completed in end-stage Kidney Disease patients, raising the need to systematically review the evidence on the association of physical activity with mortality and hospitalization in this population.

**Methods:**

Electronic databases (EBSCO, Scopus and Web of Science) and hand search were performed until March 2020 for observational studies reporting the association of physical activity with mortality or hospitalization in adult end-stage Kidney Disease patients on renal replacement therapy (hemodialysis, peritoneal dialysis and kidney transplant). Methodological quality of the included studies was assessed using the Quality in Prognosis Studies tool. The review protocol was registered in PROSPERO (CRD42020155591).

**Results:**

Eleven studies were included: six in hemodialysis, three in kidney transplant, and two in hemodialysis and peritoneal dialysis patients. Physical activity was self-reported, except in one study that used accelerometers. All-cause mortality was addressed in all studies and cardiovascular mortality in three studies. Nine studies reported a significant reduction in all-cause mortality with increased levels of physical activity. Evidence of a dose-response relationship was found. For cardiovascular mortality, a significant reduction was observed in two of the three studies. Only one study investigated the association of physical activity with hospitalization.

**Conclusions:**

Higher physical activity was associated with reduced mortality in end-stage Kidney Disease patients. Future studies using objective physical activity measures could strengthen these findings. The association of physical activity with hospitalization should be explored in future investigations.

**Supplementary Information:**

The online version contains supplementary material available at 10.1186/s12882-021-02407-w.

## Background

Progression of chronic kidney disease (CKD) is a major concern as survival of end-stage kidney disease (ESKD) patients depends on renal replacement therapy: hemodialysis (HD), peritoneal dialysis (PD) or kidney transplant (KT). Each year, about 440.000 patients worldwide start renal replacement therapy [1], with HD being the most common. However, KT is the most cost-effective, promoting a better survival and quality of life with reduced costs [2]. ESKD patients, particularly those on HD and PD, have an increased mortality and hospitalization [3], with these being important outcomes that are a priority for patients, caregivers and healthcare professionals [4, 5].

Physical activity (PA), defined as any bodily movement produced by the contraction of skeletal muscles that increases energy expenditure above basal levels [6], is associated with a reduced mortality in the general population [7]. However, a relationship between PA and mortality in ESKD has not been thoroughly established. ESKD patients have a high prevalence of traditional cardiovascular (CV) risk factors, as well as non-traditional CV risk factors, such as microalbuminuria, anaemia, oxidative stress, inflammation and hyperphosphatemia [8]. Moreover, this population has an increased risk for other specific causes of death (e.g. infection) [9]. Thus, findings related to PA and mortality in the general population cannot be extrapolated to CKD patients [10]. Several factors contribute to a vicious cycle of reduced PA in ESKD: behavioural (e.g. depression), pathophysiological (e.g. anaemia, HD-related fatigue) and logistical (e.g. time spent on dialysis) [11]. Therefore, inactivity (an insufficient PA level to meet the World Health Organization recommendations [6]) is common in ESKD patients and is associated with important predictors of mortality, such as inflammation, body composition, physical function and cardiorespiratory fitness [11]. A systematic review found an inverse association between PA and mortality in CKD [12]. However, this study was restricted to nondialysis patients, which have lower mortality and hospitalization rates compared to ESKD patients [3]. No previous systematic reviews combined the evidence on the association of PA with mortality and hospitalization in the high-risk ESKD population.

This systematic review aims to examine whether higher PA levels are associated with lower mortality and hospitalization in adult patients with ESKD on renal replacement therapy. Our summarized evidence should impact the kidney care community by raising awareness for the need to increase PA in this highly inactive population.

## Methods

### Protocol and Registration

The protocol of this study was registered on the International Prospective Register of Systematic Reviews (PROSPERO) (CRD42020155591) and can be assessed at https://www.crd.york.ac.uk/PROSPERO/. This report followed the recommendations of the Preferred Reporting Items for Systematic Reviews and Meta-Analyses guidelines [13].

### Criteria for considering studies for this review

The inclusion criteria for this review were: (1) observational studies; (2) reported associations between PA as an exposure variable with all-cause or CV mortality and/or hospitalization; (3) reported effect estimates with 95 % confidence intervals (CI); and (4) adult (age ≥ 18 years old) community-dwelling renal replacement therapy patients. Abstracts, conference papers and studies published in non-English-language journals were excluded.

An inclusive approach was used regarding PA measures. Thus, any of the following were considered: (1) any type of PA measurement, including self-reports, devices and direct observation; (2) any of the PA dimensions: frequency, intensity, time and type [14]; (3) any PA domain: leisure-time, occupational, household and transport-related [15]; (4) PA instruments reporting energy expenditure, and (5) summarized PA-related measures by devices (e.g. number of steps) [16].

Physical fitness and PA are two different constructs [17]. Thus, studies using physical fitness measures and reporting them as PA were excluded.

### Search strategy for identification of studies

The EBSCO, Scopus, and Web of Science electronic databases were searched from their date of establishment to 16 of March 2020. Text words used were: (exercise OR “physical activity”) AND (mortality OR hospital* OR “length of stay” OR “cardiovascular event”) AND (“Chronic Kidney Insufficiency” OR “Chronic Kidney Diseases” OR “Chronic Renal Diseases” OR “Chronic Renal Insufficiency” OR “Renal dialysis” OR “Hemodialysis” OR “HD” OR “Haemodialysis” OR “Kidney Transplantation” OR “Renal Transplantation” OR “Kidney Grafting”).

An additional hand search was performed to screen for other potential eligible studies including the reference lists from the included studies. Citations were managed using the software EndNote X7.3.1 (Clarivate Analytics, USA). The search strategy was conducted by one reviewer (PM). An example of a full electronic search strategy (EBSCO) can be found in additional file 1.

### Data extraction and quality assessment

Results from the overall electronic search were reviewed by two authors (PM and EAM) based on the title and abstract. Articles deemed potentially relevant were retrieved for full-text review and inspected to avoid multiple publication bias. In cases of suspected duplication, corresponding authors were contacted and, if confirmed, preference was given to the studies with a longer follow-up. Any disagreements were resolved via joint consensus.

A data extraction form was used to capture relevant information from the included studies. The following information was recorded: authors’ name, publication year, country, follow-up length, sample size, type of renal replacement therapy, participants’ mean age, sex distribution, PA measurement, PA domains, PA output, outcome measures, adjustment variables, technique for handling the missing data, and overall main findings. The exposure variable was PA and outcomes of interest were all-cause, CV or cause-specific mortality/hospitalization. Measures of association were reported in reference to the least physically active group. When needed, authors were contacted to provide the missing data.

The methodological quality of the studies was evaluated using the Quality in Prognosis Studies (QUIPS) tool [18]. This instrument includes six domains: study participation, study attrition, prognostic factor measurement, outcome measurement, study confounding, and statistical analysis and reporting. The original classification grades each domain in three categories (high, moderate or low risk of bias). We added an additional “unclear” category that should be applied when a judgement is not possible due to insufficient information. This modified QUIPS tool is described in additional file 2. Two authors (PM and EAM) assessed the risk of bias of all included studies. Discrepancies in scoring were settled by agreement.

## Results

### Search results

**Fig. 1:**
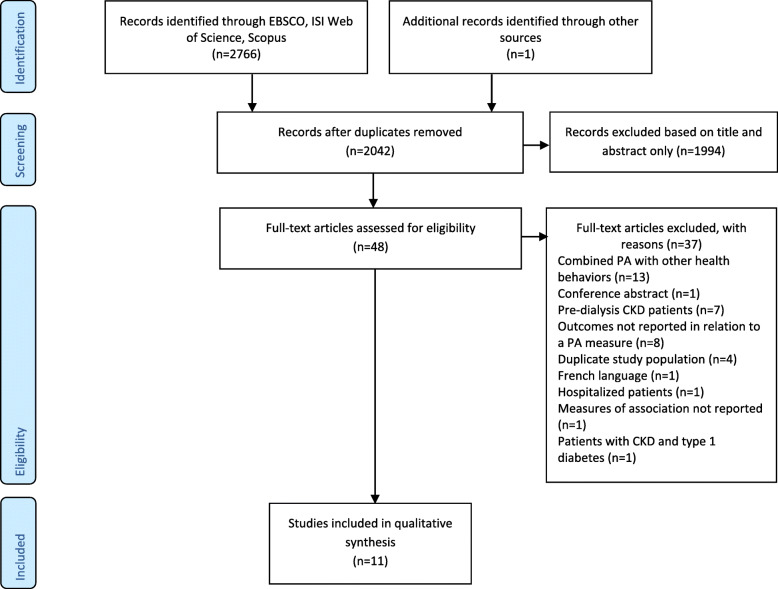
Flowchart of studies selection. *PA* physical activity; *CKD* chronic kidney disease

Electronic search retrieved 2766 records and one additional study was added through hand search [19]. After removing duplicates (n = 725), 2042 titles were screened. Whenever needed, abstracts were also analysed. Of the 48 full-text articles screened, eleven fulfilled the eligibility criteria and were included. The flow diagram of the study selection process is presented in Fig. 1, and the PRISMA checklist is presented in the additional file 3.

### Study characteristics

The characteristics of the included studies are summarized in Table 1. Four studies were from USA [22, 25, 26, 29], two from Europe [19, 28], two from Asia [23, 24] and one from Canada [27]. Two studies pooled data from several countries [20, 21]. The number of participants ranged from 109 patients [27] to 20920 patients [20], and mean age from 48 [29] to 65 years [24]. Six studies were in HD [20–25], three in KT [19, 28, 29], and two in HD and PD patients [26, 27]. None of the studies addressed PD patients alone. The mean/median length of follow-up ranged from 1.5 years [23] to 8.4 years [29]. In eight studies PA was measured using a validated self-report questionnaire [19, 21–23, 25, 27–29], two used a single question on exercise frequency [20, 26], and one study used accelerometry [24]. All the reports measured, at least, the leisure-time PA domain; one study also included the transport-related PA [19]; and three studies reported an overall score that combined all PA domains [21, 24, 28]. Zhang et al. [23] and Byambasukh et al. [19] reported outcomes in relation to specific PA intensities, namely light PA and moderate to vigorous PA, respectively.

**Table 1:** Characteristics of the included studies

Study; Country	Type of RRT; Sample size; Age (yrs); % female	Follow-up length^3^ (yrs)	PA assessment method; instrument	PA measured domains	PA (exposure) measurement scale	Outcome(s)
Tentori et al. 2010; Several countries^1^ [20]	HD; n = 20,920; 60.7 ± 14.8; 41.8	1.75	Self-reported: Single question	Leisure time	**Categorical**: never or < 1 time/wk; 1 time/wk; 2–3 times/wk; 4–5 times/wk; 6–7 times/wk (daily) **Dichotomous**: ‘regular exercise’ (≥1 time/wk) versus ‘non-regular exercise’ (never or < 1 time/wk)	All-cause mortality, All-cause hospitalization, cause-specific hospitalization
Lopes et al. 2014; Several countries^1^ [21]	HD; n = 5763; 63.4 ± 14.5; 38.3	1.6	Self-reported: RAPA	Total	**Categorical**: Never/rarely active (rarely or never do any PA); infrequently active (some light or moderate PA, not every wk); sometimes active (light PA, every wk); often active (moderate PA: < 30 min, 5 days/wk or vigorous PA: < 20 min, 3 days/wk); very active (moderate PA: > 30 min, 5days/wk or vigorous PA: > 20 min, 3days/wk)	All-cause mortality
Kutner et al. 2016; USA [22]	HD, n = 755; 57.3 ± 14.0^2^; 40.4	2.0	Self-reported: MLTAQ	Leisure time, household	**Dichotomous**: Inactive (< 500 Kcal/wk) or Active (≥ 500 Kcal/wk)	All-cause mortality
Zhang et al. 2017; China [23]	HD; n = 317; 60.2 ± 13.7; 45.4	1.5 (mean)	Self-reported: Stanford 7-PARQ	Leisure time, occupational	**Continuous**: each point increase in light PA time (hours/wk) and total PA score (kcal/kg/day)	All-cause mortality
Matsuzawa et al. 2018; Japan [24]	HD; n = 282; 64.8 ± 10.6; 45.0	4.7	Device: Accelerometer	Total	**Dichotomous**: <4000 steps/non-HD day or ≥ 4000 steps/non-HD day **Continuous**: each 1000 steps/non-HD	All-cause mortality
Johansen et al. 2019; USA; HD [25]	HD; n = 727; 57.2 ± 14.3; 40.8	3.8	Self-reported: Modified MLTAQ	Leisure time, household	**Dichotomous**: Inactive (< 383 kcal/wk; women, < 270 kcal/wk) or Active (≥ 383 kcal/wk; women, ≥ 270 kcal/wk)	All-cause mortality
Stack et al. 2005; USA [26]	HD/PD; n = 2386; 57 ± 16; 47.0	3.6 (mean)	Self-reported: Single question	Leisure time	**Categorical**: ≤1 times/wk, 2–3 times/wk, 4–5 times/wk, Daily/almost daily	All-cause mortality, CV mortality
Brar et al. 2019; Canada [27]	HD/PD; n = 109; 57.5^2^; 33.0	3.3	Self-reported PASE	Leisure time, occupational, household	**Dichotomous**: Inactive (men: <383 kcal/wk; women: <270 kcal/wk) or Active (men: ≥383 kcal/wk; women: ≥270 kcal/wk)	All-cause mortality
Zelle et al. 2011; The Netherlands [28]	KT; n = 540; 51 ± 12; 46 %	5.3	Self-reported: MLTAQ and TOAQ	Total	**Continuous**: log-MET-min/day	All-cause mortality, CV mortality
Rosas et al. 2012; USA [29]	KT; n = 507; 47.8 ± 12.8; 39 %	8.4	Self-reported: PASE	Leisure time, occupational, household	**Categorical**: inactive, moderate, active^4^ **Continuous**: PASE score	All-cause mortality
Byambasukh et al. 2020; The Netherlands [19]	KT; n = 650; 51.8 ± 13.2; 43.7 %	5.7	Self-reported: SQUASH	Leisure time, household, transportation^5^	**Categorical**: inactive (no MVPA); less active (median 120 min/wk of MVPA); active (median 360 min/wk)	All-cause mortality, CV mortality

Age is presented as mean ± standard deviation; ^1^Australia, Belgium, Canada, France, Germany, Italy, Japan, New Zealand, Spain, Sweden, UK, USA; ^2^Pooled mean based on the mean age reported for each group; ^3^median values are reported, otherwise mean values are presented as listed; ^4^Cutoffs not reported; ^5^Authors intentionally excluded occupational of the total reported PA measure

*HD* hemodialysis; *RAPA* Rapid Assessment of physical activity; *PA *physical activity; *MLTAQ *Minnesota Leisure Time Activity Questionnaire; *PARQ *Physical Activity Recall Questionnaire; *PD *peritoneal dialysis; *CV *cardiovascular; *PASE *Physical Activity Scale for the Elderly; *KT *kidney transplant; *TOAQ *Tecumseh Occupational Activity Questionnaire; *SQUASH  *Short questionnaire to assess health-enhancing physical activity; *MVPA *moderate-to-vigorous PA

All studies investigated the association of PA with all-cause mortality and, of those, three studies also investigated CV mortality [19, 26, 28]. No studies were found for other specific causes of mortality. The association between PA and hospitalization was explored in one study only [20].

### Risk of bias

Risk of bias was evaluated using the QUIPS tool (see Table 2). Across-studies, most domains showed low risk of bias. Thus, results were unlikely altered by methodological flaws. However, due to poor reporting of loss to follow up, the ‘study attrition’ domain was categorized as unclear in most studies. Potential bias in ‘prognostic factor measurement’ was related with the simplistic assessment of PA, mostly capturing only the frequency component [20, 26].

**Table 2:** Risk of bias summary of the included studies using the Quality in Prognosis Studies tool

Study	Study participation	Study attrition	Prognostic Factor Measurement	Outcome measurement	Study confounding	Statistical Analysis and Reporting
Tentori et al. 2010 [20]	Low	Low	High	Low	Low	Low
Lopes et al., 2014 [21]	Low	Unclear	Low	Low	Low	Low
Kutner et al., 2016 [22]	Low	Unclear	Low	Low	Low	Low
Zhang et al., 2017 [23]	Moderate	Low	Low	Low	Moderate	Low
Matsuzawa et al., 2018 [24]	Moderate	Unclear	Low	Low	Low	Low
Johansen et al., 2019 [25]	Low	Unclear	Low	Low	Low	Low
Stack et al., 2005 [26]	Moderate	Low	High	Low	Low	Low
Brar et al., 2019 [27]	Moderate	Low	Low	Low	Low	Low
Zelle et al., 2011 [28]	Low	Unclear	Low	Low	Low	Low
Rosas et al., 2012 [29]	Low	Unclear	Moderate	Low	Low	Low
Byambasukh et al., 2020 [19]	Low	Low	Low	Low	Low	Low

### Physical Activity and Mortality outcomes

**Fig. 2:**
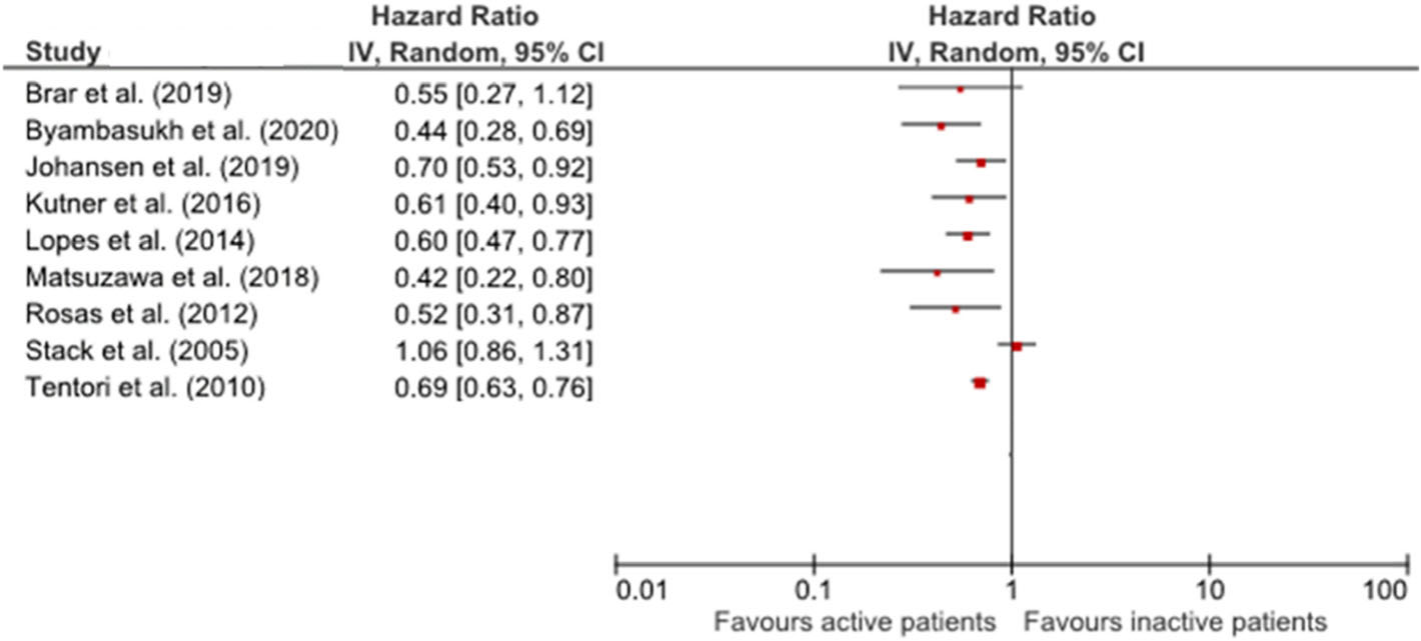
Results of the included studies comparing all-cause mortality in most active and inactive groups

Overall, the results showed that higher PA was associated with reduced mortality rates. Heterogeneity among the studies (statistically, clinically, and methodologically) precluded quantitative synthesis (meta-analysis). Hazard ratio (HR) or relative risk were reported in all studies (summarized in Table 3). For all-cause mortality, the association was statistically significant in nine studies [19–25, 28, 29]. Comparing the lowest and the highest physically active groups, HR ranged from 0.42 [95% CI: 0.22-0.82] [24] to 0.70 [95% CI: 0.53-0.93] [25] (Fig. 2). These results were independent of the exposure measure, levels for PA categorization, and adjustment for confounders. Thus, studies in HD [20–25] and KT [19, 28, 29] all supported a significant inverse association between PA and all-cause mortality. The two studies that included patients under HD or PD [26, 27] reported no evidence of significant association between mortality and PA [27], or no association only in the group of patients exercising more than 2-3 times/week [26].

**Table 3:** Summary of findings: association of PA and mortality outcomes

Study	RRT	Confounders	Main findings	Deaths n (%)	Adjusted All-cause mortality HR or RR [95 % CI]	Adjusted CV mortality HR [95 % CI]
Tentori et al. (2010) [20]	HD	Age, sex, black (Y/N), ESRD duration, BMI, 14 comorbid conditions (Y/N)^2^, albumin, phosphorus, calcium, creatinine, Hgb, catheter use (Y/N), smoker (Y/N), some college education (Y/N), employed (Y/N), private insurance (Y/N), lives alone (Y/N) and able to walk (Y/N)	All-cause mortality risk ↓ with ↑ PA (exercise frequency)	4143 (19.8)^9^	Reference: non-regular exercise (n = 10,999) Regular exercise (≥ 1 time/wk) (n = 9921): **0.73 [0.69–0.78]** Reference: <1time/wk (n = 10,999) 1 time/wk (n = 2205): **0.82 [0.73–0.91]** 2–3 times/wk (n = 3558): **0.72 [0.66–0.79]** 4–5 times/wk (n = 1201): **0.73 [0.62–0.86]** 6–7 times/wk (n = 2957): **0.69 [0.63–0.76]**	
Lopes et al. (2014) [21]		Region^3^, age, sex, black (Y/N), smoker (Y/N), employed (Y/N), some college education (Y/N), lives alone (Y/N), assistance with walking (Y/N), time on HD, strength/flexibility activities (Y/N), BMI, 14 comorbid conditions^2^, catheter use (Y/N), Hgb, Kt/V, creatinine, albumin, calcium, systolic BP < 120mmHg (Y/N), systolic BP > 160mmHg (Y/N) phosphorus, PTH and nPCR	All-cause mortality risk ↓ with ↑PA	Never/rarely active: 427 (25.9) Infrequently active: 93 (15.5) Sometimes active: 143 (14.8) Often active: 191 (13.9) Very active: 119 (10.1)	Reference: never/rarely active (n = 1649) Infrequently active (n = 599): 0.89 [0.72–1.10] Sometimes active (n = 969): 0.84 [0.67–1.05] Often active (n = 1373): **0.81 [0.68–0.96]** Very active (n = 1173): **0.60 [0.47–0.77]**	
Kutner et al. (2016) [22]		Age, sex, race (White, Black, other), college education (Y/N), current smoker (Y/N), participant clinic, BMI, diabetes (Y/N), CV comorbidity (Y/N)^4^, lupus/rheumatoid arthritis (Y/N), COPD (Y/N), cancer (Y/N), ESRD duration, catheter use (Y/N), hours wk/HD treatment	All-cause mortality risk ↓ in active patients	Inactive: 67 (18.4) Active: 43 (11.0)	Reference: inactive (n = 364) Active (n = 391): **0.61 [0.40–0.93]**	
Zhang et al. (2017) [23]		Age	All-cause mortality risk ↓ with ↑ light and overall PA	133 (42.0)^9^	Every hour/wk increase of light PA: **0.69 [0.49–0.98]** Every Kcal/kg/day increase of overall PA: **0.66 [0.45–0.95]**	
Matsuzawa et al. (2018) [24]		Age, sex, time on HD, BMI, diabetes (Y/N), peripheral vascular disease (Y/N), CBV accident/transient ischemic attack (Y/N), geriatric nutritional risk index, and comorbidity score	All-cause mortality risk ↓ with ↑steps/day	< 4000 steps/day: 61 (39.9) ≥ 4000 steps/day: 17 (13.0)	Reference: <4000 steps/day (n = 153) ≥ 4000 steps/day (n = 129): **0.42 [0.22–0.82]** Every increase of 1000 steps/day: **0.84 [0.74–0.96]**	
Johansen et al. (2019) [25]		Age, sex, race (Black, White, Asian, other), Hispanic (Y/N), BMI, time on HD, diabetes (Y/N), atherosclerotic heart disease (Y/N), heart failure (Y/N), catheter use (Y/N), albumin	All-cause mortality risk is related with all frailty components All-cause mortality risk ↓ in active patients	204 (28.1)^9^	Reference: inactive (n = 297) Active (n = 430): **0.70 [0.53–0.93]**	
Stack et al. (2005) [26]	HD + PD	Age, sex, race (White, Black, Asian), cause of ESRD (glomerulonephritis, diabetes, hypertension), congestive heart failure (Y/N), coronary artery disease (Y/N), peripheral vascular disease (Y/N), left ventricular hypertrophy (Y/N), undernourished (Y/N, caregiver subjective opinion), albumin, phosphorus and hematocrit	All-cause mortality risk ↓ for patients exercising 2–3 times/wk. No significant results for 4–5 times/wk and daily exercise. No significant results for CV mortality.	1366 (57.3)^9^	Reference: ≤1time/wk (n = 1333) 2-3times/wk (n = 437): **0.74 [0.58–0.95]** 4-5times/wk (n = 134): 0.70 [0.47–1.04] Daily (n = 482): 1.06 [0.86–1.30]	2-3times/wk: 0.80 [0.58–1.08]^1^ (Reference: ≤1time/wk)
Brar et al. 2019 [27]		Age, sex, albumin, hemoglobin and number of comorbidities	No significant reduction in all-cause mortality risk for active patients	38 (34.9)^9^	Reference: inactive Active: 0.55 [0.27–1.13]	
Zelle et al. (2011) [28]	KT	Age, sex, history of CV events^5^ (Y/N), insulin concentration, systolic BP, waist circumference, triglycerides, smoker (Y/N), CRP, Framingham risk score, creatinine clearance, urinary protein excretion, 24-h urinary creatinine	All-cause and CV mortality risk ↓ with ↑PA	81 (15.0)^9^	Every increase of 1 MET-min/day: **0.75 [0.60–0.94]**	Every increase of 1 MET-min/day: **0.62 [0.45–0.86]**
Rosas et al. (2012) [29]		Recipient and donor age, African American (Y/N), sex, diabetes (Y/N), dialysis duration, ever smoked (Y/N), BMI, delayed graft function^6^ (Y/N)	All-cause mortality risk ↓ with ↑PA at the time of kidney transplantation	Inactive: 61 (36.3) Moderate: 39 (23.3) Active: 28 (16.3)	Reference: inactive (n = 169) Moderate (n = 166): 0.87 [0.56–1.35] Active (n = 172): **0.52 [0.31–0.87]** Every 10-unit increase in PASE score: **0.96 [0.92–0.99]**	
Byambasukh et al. (2020) [19]		Age, sex, eGFR, urinary protein excretion, time between transplantation and baseline, primary renal disease^7^, acute rejection (Y/N), pre-emptive transplantation (Y/N), living donor (Y/N), current smoker (Y/N), total alcohol consumption, total energy intake, immunosuppressive medication (Y/N) ^8^, systolic BP, use of antihypertensive drugs (Y/N), triglycerides, HDL-C, BMI, waist circumference, 24-h creatinine excretion	All-cause and CV mortality risk ↓ with ↑PA	129 (19.8)^9^	Reference: inactive (n = 246) Less active (n = 201): **0.45 [0.29–0.70]** Active (n = 203): **0.44 [0.28–0.69]**	Less active: 0.55 [0.26–1.16] Active: **0.44 [0.19–0.99]** (Reference: inactive)

^1^Data not reported for other PA categories: 4-5times/wk and daily PA (results were not significant); ^2^diabetes, hypertension, coronary artery disease, congestive heart failure, other cardiovascular disease, peripheral vascular disease, cerebrovascular disease, recurrent cellulitis, GI bleed, lung disease, neurologic disorder, depression, other psychiatric disorders, cancer other than skin, HIV; ^3^Europe, Australia/New Zealand, Japan or North America; ^4^congestive heart failure, coronary artery disease, cerebrovascular accident, peripheral vascular disease, other cardiac diseases; ^5^myocardial infarction or transient ischemic attack/CBV accident; ^6^need for dialysis during the 1st week after transplantation; ^7^glomerulosclerosis, glomerulonephritis, tubulointerstitial nephritis, polycystic kidney disease, renal hypodysplasia, renavascular diseases, diabetes, others; ^8^calcineurin inhibitors, prednisolone; ^9^data not provided for each PA group

*HR* hazard ratio; *RR *relative risk; *CI *confidence interval; *CV *cardiovascular; *ESRD *end-stage renal disease; *BMI *body mass index; *BP *blood pressure; *PTH *parathyroid hormone; *nPCR *normalized protein catabolic rate; *PA *physical activity; *COPD *chronic obstructive pulmonary disease; *HD *hemodialysis; *CBV *cerebrovascular; *CRP *C-reactive protein; *MET *metabolic equivalents

Based on frequency alone [20, 26] or combining frequency, duration, and intensity [19, 21, 29], five studies grouped patients in three or more PA levels, allowing for a dose-response analysis. In four of these studies, a graduated dose-response was observed [19–21, 29]. The exception was Stack and colleagues [26] study in which the protective benefit of PA was reduced for patients exercising more than 2–3 times/week.

All studies exploring PA as a continuous variable, demonstrated that PA increments (one hour/week of light PA [23], 1000 steps/non-HD day [24], 1 MET-min/day [28], or 10-unit increase in PASE score [29]) have an inverse significant association with all-cause mortality [23, 24, 28, 29].

The association of PA with CV mortality was addressed in three studies [19, 26, 28]. Of those, two studies found an inverse significant association between PA and CV mortality with a similar magnitude to results reported for all-cause mortality [19, 28]. The other study found no evidence of association between PA and CV mortality [26].

### Physical Activity and Hospitalization outcomes

This review includes only one study addressing the association between PA and the hospitalization risk [20], which reported that patients exercising ≥ 1time/week, compared to patients exercising < 1time/week, had a similar risk of hospitalization for all-cause (HR = 1.00 [95 % CI: 0.96–1.04]), cardiac events (HR = 0.97 [95 % CI: 0.91–1.03]), and amputations (HR = 0.98, [95 % CI: 0.83–1.17]). Also, patients exercising ≥ 1time/week had a significant lower fracture-related hospitalization risk (HR = 0.76 [95 % CI: 0.61–0.94]).

## Discussion

### Summary of main findings

This systematic review found evidence of a significant and consistent association between higher PA levels and reduced mortality in ESKD patients. This finding is sustained by studies addressing all-cause mortality. Results are less robust for CV mortality as this outcome was only addressed in three studies [19, 26, 28]. The association between PA and hospitalization was only reported in one study [20], therefore no conclusions can be drawn. Nevertheless, this study demonstrated that physically active patients have a lower hospitalization risk due to fractures [20].

Results for HD and KT patients are consistent in favour of a protective effect of PA on all-cause mortality. PD patients were only investigated in combination with HD patients, and results are somewhat conflicting [26, 27]. Brar et al. [27] found a non-significant reduction in all-cause mortality. However, this is the study with the smallest sample (n = 109), possibly underpowered to find a significant association. Stack et al. [26] observed a significant lower mortality risk for patients exercising 2–3 times/week. However, unexpectedly, this benefit was reduced for patients exercising more than 2–3 times/week. Nevertheless, the large proportion of patients in the higher exercise frequency groups (e.g. 20.2 % of patients exercising daily) seems unrealistic for dialysis patients and it is indicative of some potential bias (e.g. physiotherapy) that might have led to this intriguing result. These discrepant findings suggest more research is needed, preferably with more accurate PA measures, to evaluate the dose-response association between PA and mortality.

In line with findings for the general population [7], we observed a dose-response association between PA and all-cause mortality, demonstrated in studies with three or more PA volume categories [19–21, 29], with the exception of the study from Stack et al. [26]. The evidence of a dose-response association is a relevant finding, especially for deconditioned ESKD patients since the benefits of PA were observed even with modest PA amounts.

Some methodological drawbacks can be caused by the categorization of continuous variables, particularly if data-dependent quantiles are used to form categories. Therefore, more robust findings are obtained when the exposure is analysed as a continuous variable [30]. In the present review, four studies reported results based on continuous PA data and found reductions in all-cause mortality with increments in PA [23, 24, 28, 29]. These results strengthen the evidence of an inverse association between PA and all-cause mortality in ESKD.

The association of PA with CV mortality is paramount as CV complications are the main cause of death in ESKD [1]. Although only three studies addressed this outcome [19, 26, 28], two of them found a protective role of PA that was similar for all-cause and CV mortality. This finding is in agreement to those for the general population [31]. Because in ESKD, non-CV and CV mortality share common risk factors, such as infections and inflammation [32], it is impossible to differentiate the mechanisms exclusively affecting CV from those supporting non-CV mortality. Nevertheless, several mechanisms related to PA may decrease CV mortality in ESKD. Specifically, it is known that an increase in PA may reduce the progression of atherosclerosis and even reduce the atherosclerotic burden [33], it improves endothelial function by increasing the laminar sheer stress rising nitric oxide bioavailability [34], and reduces arterial stiffness by improving vasodilation due to elevated nitric oxide levels and preventing the connective tissue building-up in the arteries [33]. PA may also exert anti-inflammatory effects [35], and has been observed to be associated with reduced oxidative stress (i.e. increase in antioxidants resulting in less accumulation of reactive oxygen species) [33]. Furthermore, higher PA levels improve CV risk profile (blood pressure control and blood lipid profile) [33], with anti-ischemic effects of PA being potentially related to an enhanced coronary blood flow reserve [33]. On the other hand, PA may activate anti-arrhythmic protective mechanisms due to a reduction in sympathetic and increase in parasympathetic/vagal stimulation of the myocardium, improving the electrical stability of the heart [33]. PA has also been observed to elicit antithrombotic effects creating an environment that favours fibrinolysis over thrombosis [33]. All these mechanisms may explain the reduced CV mortality in active patients. The prevention of muscle wasting and functional decline, important predictors of mortality in ESKD, may also explain why PA decreases all-cause mortality in this population [11].

This review provided empirical evidence that PA is associated with a reduced mortality risk in this population. Because renal replacement therapies are fundamentally used to sustain life, strategies that improve survival should be combined with this treatment. Thus, interventions to promote PA should be implemented in clinical settings.

### Strengths and limitations

Regarding the type of renal replacement therapy, none of the included studies provided data on PD patients alone, which limited our conclusions for this specific population.

Evidence shows that self-reported measures tend to underestimate the association between PA and mortality [31]. This is possibly explained by their overestimation of PA [14]. If so, we might have underestimated the protective role of PA. Also, some studies only assessed leisure-time PA, which fails to capture the overall PA behaviour. However, the resulting bias may have been minimal as this is the predominant PA domain in developed countries [36].

Another potential bias is related to the observed underreported study attrition. Thus, there may be unknown reasons for loss to follow-up that could have influenced the outcomes (e.g., discontinuation of HD treatment can precede mortality).

Additionally, reverse causality may have overestimated our results. Usually, most ill patients have reduced PA levels. Thus, the observed association between PA and mortality could also have been driven by the poor health status, rather than the PA exposure *per se* [37]. Some of the included studies have a short follow-up period and are, consequently, at an increased risk of reverse causality. Nevertheless, most studies performed an analysis adjusted for baseline comorbidities that may have minimized this bias.

Finally, confounding bias could have also influenced our results. Nevertheless, most studies performed a comprehensive adjusted analysis. Even so, we cannot rule out a residual confounding from non-controlled variables.

### Agreements and disagreements with other studies and reviews

The influence of PA on hard clinical endpoints (e.g. mortality) was already demonstrated in previous systematic reviews in the general population [7], and in patients with diabetes [38], which is commonly observed in ESKD patients. There are comparable findings to the present review. Firstly, the dose-response association between PA and all-cause mortality is supported by other reviews [7, 38]. Secondly, when inactive patients were compared with the most active patients, our observed risk reduction for all-cause mortality ranged from 30 % [25] to 58 % [24], which is comparable to that observed for diabetic patients in the same activity-pattern groups (i.e. inactive *versus* active patients) (40 %) [38]. Also, evidence suggests that PA may equally decrease mortality in healthy and in chronic disease patients [39]. However, a higher protective effect of PA (73 % reduction in all-cause mortality) has been reported for the general population [7] compared with our findings. That review only included accelerometer-based studies, which may explain the differences in the reported HR.

The mineral and bone disorders of CKD causes bone fragility resulting in a higher risk of fractures and, consequently, an increase in hospitalization and mortality rates [40]. Thus, strategies to reduce bone fractures in this population are needed. PA should be considered as a key element in these strategies, as it acts favourably in two key risk factors: bone characteristics (such as mineral density) [41] and the prevention of falls [42]. Despite in this systematic review only one study investigated the association between PA and hospitalization, a potential for a reduction in fracture-related hospitalizations is assumed [20]. This result agrees with the findings from a previous umbrella review in older adults [43].

## Conclusions

The present systematic review finds evidence of a dose-response reduction in all-cause mortality associated with increased PA. Moreover, greater PA was associated with a reduction in CV mortality; however, the lack of studies exploring the link between PA and CV mortality, as well as hospitalization, limited the strength of our conclusions.

### Implications for practice and future research

Despite the well-accepted wide-range health benefits attributed to PA, there is still a lack of efficient strategies in renal care targeting the low levels of PA in these patients [44], which may be in part because PA is a complex behaviour and its promotion is a challenge in healthcare settings [45]. Thus, to successfully change PA behaviour, approaches must be grounded in relevant theories and target both, the individual and their environment [46]. Such a comprehensive intervention is not common in ESKD patients, but is popular in pulmonary [47] and cardiac rehabilitation [48]. Nevertheless, some successful approaches of sustainable exercise programs are also known for ESKD patients, including intradialytic exercise, home-based exercise, inter-dialytic exercise, prehabilitation for transplant candidates, rehabilitation post-transplant and education programs [49]. The present findings should inform policy makers and other stakeholders for the need to address inactivity in this population.

We identified several issues that must be a priority in future studies design, specifically (1) targeting all different renal replacement therapy (particularly PD patients), (2) collecting data on the specific causes of mortality (e.g., CV, infection, or cancer) and in hospitalization outcomes, and (3) using accelerometry to objectively measure PA. This would provide more reliable PA data and the opportunity to explore the role of each PA components (frequency, intensity, and duration), and sedentary behaviour. Moreover, collecting PA data at different timepoints could inform the clinical significance of PA variations over time.

## Supplementary Information

Additional file 1 Search strategy used for EBSCO.

Additional file 2 Quality assessment of the included studies (adapted Quality in Prognosis Studies tool).

Additional file 3 PRISMA Checklist. Twenty-seven-item checklist reporting of a systematic review.

## Data Availability

The datasets generated during and/or analyzed during the current study are available from the corresponding author on reasonable request.

## Abbreviations

CI: Confidence Interval
CKD: Chronic Kidney Disease
CV: Cardiovascular
ESKD: End-stage Kidney Disease
HD: Hemodialysis
HR: Hazard Ratio
KT: Kidney Transplant
PA: Physical Activity
PD: Peritoneal Dialysis
QUIPS: Quality in Prognosis Studies
