# Supplementary material for: Association between physical activity and mortality in end-stage kidney disease: a systematic review of observational studies
Source: BMC Nephrol. 2021 Jun 18;22:227. doi: 10.1186/s12882-021-02407-w (PMC8212466; doi:10.1186/s12882-021-02407-w)
Supplement: Supplementary file 1 — Search strategy used for EBSCO. [file 12882_2021_2407_MOESM1_ESM.docx]

**Supplementary material 1.**

**Search strategy used for EBSCO**

**Query Translation**

(“exercise” [All Fields] OR “physical activity” [All Fields]) AND (“mortality” [All Fields] OR “hospital*” [All Fields] OR “length of stay” [All Fields] OR “cardiovascular event” [All Fields]) AND (“Chronic Kidney Insufficiency” [All Fields] OR “Chronic Kidney Diseases” [All Fields] OR “Chronic Renal Diseases” [All Fields] OR “Chronic Renal Insufficiency” [All Fields] OR “Renal dialysis” [All Fields] OR “Hemodialysis” [All Fields] OR “HD” [All Fields] OR “Haemodialysis” [All Fields] OR “Kidney Transplantation” [All Fields] OR “Renal Transplantation” [All Fields] OR “Kidney Grafting” [All Fields]).

**Individual translations**

| Physical activity | “exercise” [All Fields] OR “physical activity” [All Fields] |
| --- | --- |
| Mortality | “mortality” [All Fields] OR “cardiovascular event” [All Fields] |
| Hospitalization | “hospital*” [All Fields] OR “length of stay” [All Fields] |
| End-stage renal disease | “Chronic Kidney Insufficiency” [All Fields] OR “Chronic Kidney Diseases” [All Fields] OR “Chronic Renal Diseases” [All Fields] OR “Chronic Renal Insufficiency” [All Fields] OR “Renal dialysis” [All Fields] OR “Hemodialysis” [All Fields] OR “HD” [All Fields] OR “Haemodialysis” [All Fields] OR “Kidney Transplantation” [All Fields] OR “Renal Transplantation” [All Fields] OR “Kidney Grafting” [All Fields] |
