# Supplementary material for: Association between physical activity and mortality in end-stage kidney disease: a systematic review of observational studies
Source: BMC Nephrol. 2021 Jun 18;22:227. doi: 10.1186/s12882-021-02407-w (PMC8212466; doi:10.1186/s12882-021-02407-w)
Supplement: Supplementary file 2 — Quality assessment of the included studies (adapted Quality in Prognosis Studies tool). [file 12882_2021_2407_MOESM2_ESM.docx]

**Quality assessment of the included studies (adapted Quality in Prognosis Studies tool)**

| **Domains** | **Prompting items for Consideration (Yes, No, N/A)** | **Rating (Low, Moderate, High, Unclear)** | **Observations** |
| --- | --- | --- | --- |
| Study participation | - Adequate participation in the study by eligible persons - Description of the source population or population of interest - Description of the baseline study sample - Adequate description of the sampling frame and recruitment - Adequate description of the period and place of recruitment - Adequate description of inclusion and exclusion criteria | **High bias:** The relationship between the PF and outcome is very likely to be different for participants and eligible nonparticipants  **Moderate bias**: The relationship between the PF and outcome may be different for participants and eligible nonparticipants  **Low bias**: The relationship between the PF and outcome is unlikely to be different for participants and eligible nonparticipants  **Unclear**: insufficient information |  |
| Study attrition | - Adequate response rate for study participants - Description of attempts to collect information on participants who dropped out - Reasons for loss to follow-up are provided - Adequate description of participants lost to follow-up - There are no important differences between participants who completed the study and those who did not | **High bias**: The relationship between the PF and outcome is very likely to be different for completing and non-completing participants  **Moderate bias**: The relationship between the PF and outcome may be different for completing and non-completing participants  **Low bias**: The relationship between the PF and outcome is unlikely to be different for completing and non-completing participants  **Unclear**: insufficient information |  |
| Prognostic Factor Measurement | - A clear definition or description of the PF is provided - Method of PF measurement is adequately valid and reliable - Continuous variables are reported or appropriate cut points are used - The method and setting of measurement of PF is the same for all study participants - Adequate proportion of the study sample has complete data for the PF - Appropriate methods of imputation are used for missing PF data | **High bias**: The measurement of the PF is very likely to be different for different levels of the outcome of interest  **Moderate bias**: The measurement of  the PF may be different for different levels of the outcome of interest  **Low bias**: The measurement of the PF is unlikely to be different for different levels of the outcome of interest  **Unclear**: insufficient information |  |
| Outcome measurement | - A clear definition of the outcome is provided - Method of outcome measurement used is adequately valid and reliable - The method and setting of outcome measurement are the same for all study participants | **High bias**: The measurement of the outcome is very likely to be different related to the baseline level of the PF  **Moderate bias**: The measurement of the outcome may be different related to the baseline level of the PF  **Low bias**: The measurement of the outcome is unlikely to be different related to the baseline level of the PF  **Unclear**: insufficient information |  |
| Study confounding | - All-important confounders are measured - Clear definitions of the important confounders measured are provided - Measurement of all-important confounders is adequately valid and reliable - The method and setting of confounding measurement are the same for all study participants - Appropriate methods are used if imputation is used for missing confounder data - Important potential confounders are accounted for in the study design - Important potential confounders are accounted for in the analysis | **High bias:** The observed effect of the PF on the outcome is very likely to be distorted by another factor related to PF and outcome  **Moderate bias:** The observed effect of the PF on outcome may be distorted by another factor related to PF and outcome  **Low bias:** The observed effect of the PF on outcome is unlikely to be distorted by another factor related to PF and outcome  **Unclear**: insufficient information |  |
| Statistical Analysis and  Reporting | - Sufficient presentation of data to assess the adequacy of the analytic strategy - Strategy for model building is appropriate and is based on a conceptual framework or model - The selected statistical model is adequate for the design of the study - There is no selective reporting of results | **High bias**: The reported results are very likely to be spurious or biased related to analysis or reporting  **Moderate bias**: The reported results may be spurious or biased related to analysis or reporting  **Low bias**: The reported results are unlikely to be spurious or biased related to analysis or reporting  **Unclear**: insufficient information |  |
